# Epidemiological Study of Lactose- and Non-lactose-Fermenting Gram-Negative Bacterial Isolates From Clinical Specimens in the Middle Al-Furat Region of Iraq

**DOI:** 10.7759/cureus.93461

**Published:** 2025-09-29

**Authors:** Ehsan A Alaltoganee, Hayder A Muhammed

**Affiliations:** 1 Department of Health Sciences, Faculty of Medicine “Ibn El Jazzar” of Sousse, Sousse, TUN; 2 Department of Microbiology, Faculty of Veterinary Medicine, University of Kerbala, Kerbala, IRQ

**Keywords:** antibiotics pathogen, lactose fermentation g-ve bacteria, multidrug resistant (mdr), non-lactose fermentation g-ve bacteria, nosocomial epidemiology

## Abstract

Background: The study included a collection of 682 clinical samples from the hospital laboratories all over Iraq's Middle Euphrates region, which encompasses the governorates of Najaf, Karbala, Babylon, and Diwaniyah. The study lasted for seven months, from April 2022 to the end of October 2022, and aimed to isolate and diagnose Gram-negative lactose-fermentative bacterial species while determining the extent of multidrug resistance (MDR) of the isolated bacteria to antibiotics.

Method: Swabs were quickly taken to the lab in a special solution and placed on different types of growth media, like blood agar, nutrition agar, and MacConkey agar, and then the Vitek Compact 2 system was also used to confirm the identification of the bacteria. Susceptibility to antimicrobials was assessed according to the CLSI guideline.

Results: Six hundred and eighty-two Gram-negative bacterial isolates revealed that 468 (68.6%) were lactose-fermenting species, including E. coli (47.7%, 325/682), Klebsiella pneumoniae (13.9%, 95/682), and Enterobacter cloacae (7.0%, 48/682), while 214 (31.4%) were non-lactose-fermenters, predominantly Pseudomonas aeruginosa (10.6%, 72/682), Acinetobacter baumannii (8.5%, 58/682), Salmonella typhi (8.9%, 61/682), and Proteus mirabilis (3.4%, 23/682). Antibiotic susceptibility testing showed the highest efficacy for ampicillin (84.2%) and amoxicillin/clavulanic acid (75.5%), followed by cefotaxime (75.5%), ceftazidime (71%), imipenem (58.4%), and meropenem (63.8%), with all isolates exhibiting significant resistance (p < 0.0001)

Conclusion: This work emphasizes the place of the Gram-negative bacteria as nosocomial and community-acquired pathogens. The rates of resistance are in accordance with a public health problem in the Middle Euphrates region.

## Introduction

Healthcare institutions are profoundly concerned about the prevalence of urinary tract infections (UTIs), which rank among the most common infections in human medicine. The clinical microbiology laboratory receives more urine specimens for culture than any other sample type, and there exists a strong correlation between the investigated patient population and the patterns of uropathogen susceptibility, species distribution, and epidemiology, which vary significantly over time and geography [1]. The increasing incidence of bacterial illnesses, particularly those attributed to lactose-fermentative and non-lactose-fermentative bacteria, constitutes a significant public health concern globally, including in the Middle Euphrates region of Iraq. Bacterial isolates from clinical specimens, including urine, blood, sputum, and wound swabs, are pivotal in both hospital-acquired and community-acquired infections, exhibiting diverse levels of antimicrobial resistance (AMR) and clinical outcomes [2].

Enterobacteriaceae bacteria, especially Escherichia coli and Klebsiella spp., are the most clinically relevant lactose-fermenting (LF) bacteria and are often linked to UTIs, bloodstream infections, and pneumonia [3]. Microbiological diagnostics like MacConkey agar differentiation use these bacteria's lactose fermentation. Non-lactose fermentative bacteria, such as Pseudomonas aeruginosa, Proteus spp., Salmonella spp., and Acinetobacter baumannii, are equally important pathogens but harder to treat due to their intrinsic antibiotic resistance and tendency to cause severe nosocomial infections [4]. Due to their different resistance patterns and clinical symptoms, LF and non-lactose fermenting (NLF) bacteria must be distinguished for laboratory identification and epidemiological surveillance [5]. Healthcare-associated infections are rising in Iraq, especially in Middle Euphrates cities like Najaf, Karbala, Babil, and Al-Qadisiyyah, with more antibiotic-resistant strains likely caused by overuse of antibiotics, poor infection control, and a lack of diagnostic tools [6]. Tracking LF and NLF bacteria's prevalence, resistance to treatment, and health effects can help shape treatment plans and public health actions. Their ability to cause disease, harmful traits, and resistance genes affects how serious the illness is and how well treatments work [7]. The most prevalent LF bacterium, E. coli, causes UTIs and sepsis. Extended-spectrum beta-lactamase (ESBL) bacteria are becoming more common in Iraqi hospitals, complicating treatment. Klebsiella pneumoniae, another LF bacterium, often develops carbapenem resistance, making infections difficult to treat. In contrast, NLF bacteria like Pseudomonas aeruginosa can expel drugs, develop biofilms, and rapidly evolve, making them chronic concerns in ICUs and burn units. Other NLF pathogens, including Acinetobacter spp., cause ventilator-associated pneumonia (VAP) and wound infections, while growing multidrug-resistant (MDR) strains restrict treatment [8]. Given these challenges, clinical specimen bacterial isolates must be monitored to identify resistance trends and implement effective antibiotic stewardship programs [9].

The Middle Euphrates region of Iraq has a varied healthcare infrastructure, including insufficient laboratory capacity, which delays bacterial identification and susceptibility testing. Diagnostic gaps may lead to empirical antibiotic usage, worsening resistance [10]. Local studies are needed to determine the impact of LF and NLF bacteria since income, population, and sanitation affect bacterial infection dissemination [11]. There is little data on the Middle Euphrates governorates, but Iraqi studies have shown an increase in MDR pathogens [12]. The distribution of these bacteria in urine, blood, and respiratory secretions can reveal their transmission dynamics and assist infection prevention [13]. Resistant infections that emerge in the community may indicate bigger public health concerns that require care beyond hospitals [14].

This study aims to isolate and identify Gram-negative enteric bacteria based on their ability to ferment lactose and to subsequently determine their antibiotic resistance profiles.

## Materials and methods

Size of the study's sample and population

A cross-sectional study analyzed all consecutive clinical samples (682) collected from hospital laboratories across four governorates in Iraq's Middle Euphrates region (Najaf, Karbala, Babylon, and Diwaniyah) between April and October 2022. There were 682 patients in this study: 279 male patients and 403 female patients. They range in age from 10 to 70 years, and they all had conditions like foot ulcers, otitis media, orchitis, respiratory tract infections, UTIs, vaginitis, burns, sepsis, and meningitis. Participants were not given antibiotics in the 3-5 days before sampling due to their effect on bacterial culture results.

Inclusion criteria

Inclusion criteria are as follows: (i) No antibiotic exposure 72 hours prior to sampling, (ii) Clinically significant infection (fever + localizing symptoms), and (iii) First isolate per patient.

Exclusion criteria

Exclusion criteria are as follows: (i) Immunocompromised patients (HIV/AIDS, active chemotherapy, transplant recipients), (ii) Non-sterile site colonization, and (iii) Repeat samples from chronic infections. Samples were collected aseptically, transported in nutrient broth, and processed within two hours. Isolates were cultured on blood/MacConkey/nutrient agars, with identification/susceptibility testing via Vitek Compact 2 (CLSI guidelines 2023).

Laboratory diagnosis of bacterial isolation and identification focused on Gram-negative bacteria

Within an hour of collection, the swabs were transferred to a transport medium for further processing. They were subsequently cultivated on different media, such as blood agar (Oxoid), nutrition agar (Germany), and MacConkey agar (HiMedia). Following this, they were incubated aerobically at 37°C for 24 to 48 hours, and traditional biochemical tests, such as indol, methyl red, Voges Proskauer, Simon citrate, catalase, and triple sugar iron slant for the reaction pattern, followed by tests using triple sugar iron agar and certain bacterial species, are used for isolating a single colony based on culture, morphology, and biochemical test characteristics [15]. The bacteria were suspended in 3.0 mL of 0.45% saline solution in a 12 x 75 mm transparent polystyrene test tube after a sterile brush or applicator stick had transferred enough pure culture colonies. After making the necessary adjustments, the turbidity was measured using a DensiChek™ turbidity meter (bioMérieux SA, Marcy-l'Étoile, France). Vitek-2 automated reader (bioMérieux SA, Marcy-l'Étoile, France)-incubator cards were inoculated with suspension vials and loaded [16].

Antibiotic susceptibility test

The tests were performed on Muller-Hinton agar (HiMedia) according to the Clinical Laboratory Standards Institute (CLSI 2023) guidelines, using antibiotic disks. The following antibiotics were tested by Mast Diagnostics: Eight β-lactam antibiotics (ampicillin, amoxicillin/clavulanic acid, piperacillin/tazobactam, cefotaxime, ceftazidime, cefepime, imipenem, and meropenem) and eight non-β-lactam antibiotics (tetracycline, gentamicin, amikacin, nalidixic acid, fosfomycin, levofloxacin, azithromycin, and trimethoprim/sulfamethoxazole) were used from Oxoid. The bacteria should be suspended in saline for a brief period of time until the turbidity reaches 0.5 McFarland standards. The double disc synergy test using cefotaxime (30 μg) + ceftazidime (30 μg) disks with an amoxicillin-clavulanate (20/10 μg) center disk (UK) was used to identify Gram-negative bacteria (LF and NLF) that produce ESBL [17]. The researchers ascertained the minimum inhibitory concentrations for colistin using broth microdilution, following the guidelines set out by the Clinical and Laboratory Standards Institute (CLSI 2023), and then inoculated the bacterial suspensions using swabs and a wire loop. It was spread out on a plate of Mueller Hinton agar (MHA) and let to dry at room temperature for a while [18]. The next step was to stack antibiotic pills on top of agar, which was then incubated at 35°C for 18 to 24 hours. After removing the colony from the culture media, it was transferred to a glass tube that held the salt water; therefore, after carefully positioning the specimen test tubes in their designated positions, the cassette was fed into the 'Filler Station.' The cassette was received by the Vitek 2 Compact GN-ID and AST-N220 cards, which follow CLSI 2023 guidelines (“bioMérieux, France), and the loading station within 10 minutes [18].

Data management and statistical analysis

We verified the accuracy and consistency of the data from both the patients and the controls using IBM SPSS Statistics for Windows, Version 28 (Released 2021; IBM Corp., Armonk, New York, United States) before converting it into a computerized dataset. Types of variables are used to summarize the data; for example, percentages and frequencies are used for qualitative variables, and for chi-square, interquartile range, lowest, and maximum values are all displayed for scale (continuous) variables. The Kolmogorov-Smirnov test was used to ensure that the scale (continuous) variables followed a normal distribution before the statistical analysis was started.

## Results

Table 1 presents the age and gender distribution of patients with a specific type of disease caused by local site infections in the public hospital located in the Middle Al-Furat region. In this study, we found that the infection rate among 682 patients, which included 403 female patients, was higher than that among 279 male patients suffering from various diseases. Table 1 explains the distribution of infection. It displays the infections in descending order from the most severe to the least severe, including UTI (386; 56.6%), respiratory tract infection (RTI) (82; 12%), burn (61; 8.9%), sepsis (50; 7.4%), vaginitis (50; 7.4%), intestinal infection (37; 5.4%), orchitis (7; 1%), otitis media (5; 0.7%), and meningitis (4; 0.6%). This data indicates that female patients have a higher rate of urinary tract infections, with female patients accounting for 403 cases (59%) compared to male patients, who account for 279 cases (41%). There were significant differences (P < 0.05) in all types of infection and gender. The overall p-value < 0.05 for the combined dataset also confirms that gender is a statistically significant factor in infection distribution across all types. This consistent significance implies that gender-related biological, behavioral, or environmental factors could influence susceptibility to different infections. 

**Table 1:** The distribution of types of infection according to gender RTI: Respiratory tract infection; UTI: urinary tract infection

Type of infection	Gender		Total	Percentage	p-value
	M	F			
Burn	16	45	61	8.9%	< 0.05
Intestinal infection	36	1	37	5.4%	
Meningitis	3	1	4	0.6%	
Orchitis	7	0	7	1%	
Otitis media	4	1	5	0.7%	
RTI	22	60	82	12%	
Sepsis	21	29	50	7.4%	
UTI	170	216	386	56.6%	
Vaginitis	0	50	50	7.4%	
Total	279	403	682	100%	

**Figure 1:**
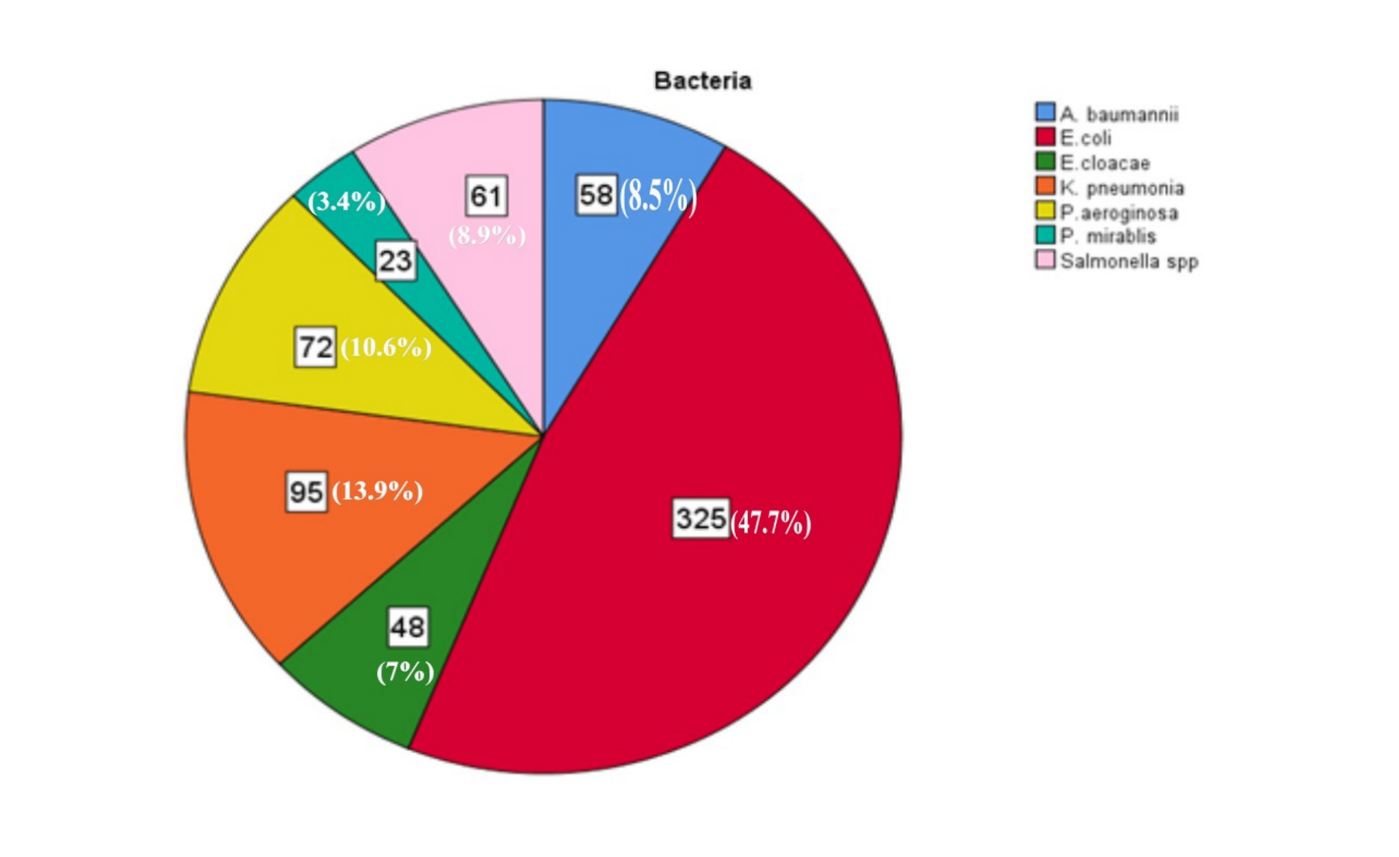
The distribution of the higher bacteria spp

Table 2 shows that the samples were isolated and transferred to the microbiological laboratory, which consisted of two groups: first, Gram-negative LF bacteria were E. coli (325; 47.7%), Klebsiella pneumoniae (95; 13.9%), and Enterobacter cloacae (48; 7.0%), while the NLF bacteria were Pseudomonas aeruginosa (72; 10.6%), Salmonella typhi (61; 8.9%), Acinetobacter baumannii (58; 8.5%), and Proteus mirabilis (23; 3.4%) (Table 2 and Figure 1).

**Table 2:** Distribution of the bacteria in the body site infection RTI: Respiratory tract infection; UTI: urinary tract infection

Type of infection	A. baumannii	E.coli	E.cloacae	K. pneumonia	P. aeroginosa	P. mirablis	Salmonella spp
Burn	31 (4.5%)	2 (0.3%)	0 (0%)	5 (0.7%)	23 (3.4%)	0 (0%)	0 (0%)
Intestinal infection	0 (0%)	0 (0%)	0 (0%)	0 (0%)	0 (0%)	0 (0%)	37 (5.4%)
Meningitis	0 (0%)	1 (0.1%)	0 (0%)	0 (0%)	3 (0.4%)	0 (0%)	0 (0%)
Orchitis	0 (0%)	0 (0%)	1 (0.2%)	6 (0.8 %)	0 (0%)	0 (0%)	0 (0%)
Otitis media	1 (0.1%)	1 (0.1%)	0 (0%)	0 (0%)	3 (0.4%)	0 (0%)	0 (0%)
RTI	4 (0.6%)	2 (0.3%)	9(1.3%)	54 (7.9%)	9 (1.3%)	4 (0.6%)	0 (0%)
Sepsis	7 (1%)	12 (1.8%)	0 (0%)	0 (0%)	3 (0.4%)	4 (0.6%)	24 (3.5%)
UTI	15 (2.2%)	274 (40.2%)	36 (5.3%)	16 (2.3%)	30 (4.4%)	15 (2.2%)	0 (0%)
Vaginatis	0 (0%)	33 (4.8%)	2 (0.3%)	14 (2.1%)	1 (0.1%)	0 (0%)	0 (0%)
Total	58 (8.5%)	325 (47.7%)	48 (7%)	95 (13.9%)	72 (10.6%)	23 (3.4%)	61 (8.9%)

Table 3 shows the total of 682 samples of Gram-negative bacteria that either ferment lactose or do not, along with their characteristics on MacConkey agar. Antimicrobial resistance testing demonstrated critical resistance patterns among Gram-negative isolates, with A. baumannii showing 98.3% resistance (57/58 isolates) to cefotaxime, the highest among all species tested. Overall resistance was most severe for ampicillin (84.2%, 574/682), amoxicillin/clavulanate (75.5%, 515/682), and third-generation cephalosporins (cefotaxime 75.5%, ceftazidime 71.0%). Carbapenem resistance was alarmingly high (imipenem 58.4%, meropenem 63.8%), though P. aeruginosa retained moderate susceptibility to meropenem (49.3%, 32/65 susceptible). E. coli dominated absolute resistance counts (e.g., 284/325 isolates resistant to ampicillin, 87.4%), while Salmonella spp. showed unexpectedly high fluoroquinolone resistance (nalidixic acid 80.3%, 49/61). Amikacin showed the best overall activity (51.2% susceptibility, 349/682), particularly against P. aeruginosa (73.8% susceptible, 48/65). All resistance patterns were statistically significant (p<0.05) except imipenem (p=0.209).

**Table 3:** Ability of the Gram-negative lactose and non-lactose fermentation bacterial isolates to ward off the antibiotic resistance ampicillin (84.2%), amoxicillin/clavulanic acid (75.5%), piperacillin/tazobactam (57.6%), cefotaxime (75.5%), ceftazidime (71%), cefepime (51.6%), imipenem (58.4%), meropenem (63.8%), amikacin (48.8%), gentamicin (60.4%), ciprofloxacin (69.4%), fosfomycin (63%), levofloxacin (55.3%), nalidixic acid (66.6%), azithromycin (58.2%), tetracycline (68.6%), and trimethoprim/sulfamethoxazole (60%)

Antimicrobial resistance	No. (%)	A. baumannii	E. coli	E. cloacae	K. pneumoniae	P.aeruginosa	P.mirabilis	Salmonella spp	Total	p-value
Ampicillin	R	52 (7.6%)	284 (41.6%)	29 (4.3%)	92 (13.5%)	69 (10.1%)	17 (2.5%)	31 (4.5%)	574 (84.2%)	< 0.001
	S	6 (0.9%)	41 (6%)	19 (2.8%)	3 (0.4%)	3 (0.4%)	6 (0.9%)	30 (4.4%)	108 (15.8%)	
Amoxicillin/Clavulanic Acid	R	51 (7.5%)	275 (40.3%)	26 (3.8%)	81 (11.9%)	48 (7%)	11 (1.6%)	23 (3.4%)	515 (75.5%)	< 0.001
	S	7 (1%)	50 (7.3%)	22 (3.2%)	14 (2.1%)	24 (3.5%)	12 (1.8%)	38 (5.6%)	167 (24.5%)	
Piperacillin/Tazobactam	R	29 (4.3%)	194 (28.4%)	19 (2.8%)	51 (7.5%)	42 (6.2%)	10 (1.5%)	48 (97%)	393 (57.6%)	< 0.001
	S	29 (4.3%)	131 (19.2%)	29 (4.3%)	44 (6.5%)	30 (4.4%)	13 (1.9%)	13 (1.9%)	289 (42.4%)	
Cefotaxime	R	57 (8.4%)	242 (35.5%)	30 (4.4%)	69 (10.1%)	62 (9.1%)	14 (2.1%)	41 (6%)	515 (75.5%)	< 0.001
	S	1 (0.1%)	83 (12.2%)	18 (2.6%)	26 (3.8%)	10 (1.5%)	9 (1.3%)	20 (2.9%)	167 (24.5%)	
Ceftazidime	R	53 (7.8%)	230 (33.7%)	30 (4.4%)	67 (9.8%)	62 (9.1%)	16 (2.3%)	26 (3.8%)	484 (71%)	< 0.001
	S	5 (0.7%)	95 (13.9%)	18 (2.6%)	28 (4.1%)	10 (1.5%)	7 (1%)	35 (5.1%)	198 (29%)	
Cefepime	R	42 (6.2%)	164 (24%)	25 (3.7%)	47 (6.9%)	57 (8.4%)	4 (0.6%)	13 (1.9%)	352 (51.6%)	< 0.001
	S	16 (2.3%)	161 (23.6%)	23 (3.4%)	48 (7%)	15 (2.2%)	19 (2.8%)	48 (7%)	330 (48.4%)	
Imipenem	R	33 (4.8%)	191 (28%)	25 (3.7%)	65 (9.5%)	40 (5.9%)	15 (2.2%)	29 (4.3%)	398 (58.4%)	0.209
	S	25 (3.7%)	134 (19.6%)	23 (3.4%)	30 (4.4%)	32 (4.7%)	8 (1.2%)	32 (4.7%)	284 (41.6%)	
meropenem	R	38 (5.6%)	199 (29.2%)	27 (4%)	68 (10%)	46 (6.7%)	21 (3.1%)	36 (5.3%)	435 (63.8%)	0.04
	S	20 (2.9%)	126 (18.5%)	21 (3.1%)	27 (4%)	26 (3.8%)	2 (0.3%)	25 (3.7%)	247 (36.2%)	
Amikacin	R	45 (6.6%)	169 (24.8%)	26 (3.8%)	33 (4.8%)	24 (3.5%)	9 (1.3%)	27 (4%)	333 (48.8%)	< 0.001
	S	13 (1.9%)	156 (22.9%)	22 (3.2%)	62 (9.1%)	48 (7%)	14 (2.1%)	34 (5%)	349 (51.2%)	
Gentamicin	R	42 (6.2%)	211 (30.9%)	26 (3.8%)	43 (6.3%)	41 (6%)	7 (1%)	42 (6.2%)	412 (60.4%)	< 0.001
	S	16 (2.3%)	114 (16.7%)	22 (3.2%)	52 (7.6%)	31 (4.5%)	16 (2.3%)	19 (2.8%)	270 (39.6%)	
Ciprofloxacin	R	52 (7.6%)	237 (34.8%)	35 (5.1%)	60 (8.8%)	31 (4.5%)	13 (1.9%)	45 (6.6%)	473 (69.4%)	< 0.001
	S	6 (0.9%)	88 (12.9%)	13 (1.9%)	35 (5.1%)	41 (6%)	10 (1.5%)	16 (2.3%)	209 (30.6%)	
Fosfomycin	R	44 (6.5%)	230 (33.7%)	28 (4.1%)	43 (6.3%)	39 (5.7%)	7 (1%)	39 (5.7%)	430 (63%)	< 0.001
	S	14 (2.1%)	95 (13.9%)	20 (2.9%)	52 (7.6%)	33 (4.8%)	16 (2.3%)	22 (3.2%)	252 (37%)	
Levofloxacin	R	46 (6.7%)	205 (30.1%)	22 (3.2%)	50 (7.3%)	22 (3.2%)	7 (1%)	25 (3.7%)	377 (55.3%)	< 0.001
	S	12 (1.8%)	120 (17.6%)	26 (3.8%)	45 (6.6%)	50 (7.3%)	16 (2.3%)	36 (5.3%)	305 (44.7%)	
Nalidixic Acid	R	48 (7%)	232 (34%)	29 (4.3%)	52 (7.6%)	38 (5.6%)	6 (0.9%)	49 (7.2%)	454 (66.6%)	< 0.001
	S	10 (1.5%)	93 (13.6%)	19 (2.8%)	43 (6.3%)	34 (5%)	17 (2.5%)	12 (1.8%)	228 (33.4%)	
Azithromycin	R	45 (6.6%)	197 (28.9%)	26 (3.8%)	45 (6.6%)	34 (5%)	9 (1.3%)	41 (6%)	397 (58.2%)	<0.001
	S	13 (1.9%)	128 (18.8%)	22 (3.2%)	50 (7.3%)	38 (5.6%)	14 (2.1%)	20 (2.9%)	285 (41.8%)	
Tetracycline	R	49 (7.2%)	276 (40.5%)	24 (3.5%)	47 (6.9%)	31 (4.5%)	10 (1.5%)	31 (4.5%)	468 (68.6%)	< 0.001
	S	9 (1.3%)	49 (7.2%)	24 (3.5%)	48 (7%)	41 (6%)	13 (1.9%)	30 (4.4%)	214 (31.4%)	
Trimethoprim/Sulfamethoxazole	R	50 (7.3%)	198 (29%)	29 (4.3%)	62 (9.1%)	32 (4.7%)	7 (1%)	31 (4.5%)	409 (60%)	< 0.001
	S	8 (1.2%)	127 (18.6%)	19 (2.8%)	33 (4.8%)	40 (5.9%)	16 (2.3%)	30 (4.4%)	273 (40%)	

The study also found that NLF Gram-negative bacteria responded differently to some antibiotics: A. baumannii was 94% effective with cefotaxime, Proteus was 74% effective with amoxicillin, Salmonella typhi was 80% effective with nalidixic acid, and P. aeruginosa was 96% effective with cefotaxime. The antibiotics that worked well included piperacillin/tazobactam at 51% for A. baumannii, levofloxacin at 83% for Proteus, fosfomycin at 70% for Salmonella typhi, and fosfomycin at 71% for P. aeruginosa. The results indicated that there were significant differences (p < 0.05) in how these bacteria responded to all the antibiotic treatments.

## Discussion

Table 1 shows that since the urethra is near the rectum in women, bacteria from the vaginal area can penetrate the urinary tract; also, the external urethral meatus in women is mostly made of mucosa (the moist tissue that lines the vagina), which is thinner and more sensitive than regular skin, helping to explain why women develop UTIs more often [19]. The higher number of UTIs in women can be explained by a few reasons, including that women's urethras are much shorter than men's, and the external urethral meatus in women is made of more sensitive mucosal tissue, which is thinner and more delicate than most skin on the body [20]. The significantly higher infection rate among women (59% vs. 41%) aligns with regional studies from Iraq [21] and Saudi Arabia [22], where UTIs, accounting for 56.6% of our cases, were 2-3 times more prevalent in women due to anatomical and behavioral factors. The dominance of E. coli is evident. E. coli (47.7%) mirrors surveillance data from Baghdad (47%) and Tehran (52%) [23,24], suggesting consistent uropathogen profiles across the Middle East. The high UTI prevalence among female patients (56.6% of infections) aligns with established anatomical risk factors: shorter urethral length and mucosal tissue vulnerability facilitating enteric pathogen entry [17,19]. E. coli dominated UTIs (40.2% of isolates), consistent with Iraqi surveillance data where it accounted for 44-52% of uropathogens [25].

The female urethra's close proximity to the rectum further enhances the transfer of bacteria. Additionally, sexual activity can introduce bacteria from the vagina into the urethra. The irritation caused by substances such as fertilizers or diaphragms, along with changes that occur during menopause and pregnancy, increases women's likelihood of developing infections due to hormonal changes (low estrogen) and aging connective tissue. Furthermore, issues like pelvic organ prolapse and incontinence are becoming more common, which leads to a higher incidence of UTIs in women who are going through menopause or after it [20]. Urinary incontinence complicates hygiene for perimenopausal women, while the thinning of the vaginal lining raises the risk of vaginal infections that may spread to the urinary system; these factors contribute to a higher likelihood of UTIs, and the findings of this study are comparable to those observed in younger women prior to menopause [21]. Moreover, it is concurred that a UTI is among the most common diseases affecting women. UTIs can originate in the alimentary canal and often coexist with vaginal infections, and this study looks at the frequency of UTIs in different patient populations, such as women going through menopause, pregnant women, diabetics, epileptics, and those who are undergoing surgery [22].

Table 2 shows that E. coli is the most common infection that causes UTIs because the bacteria usually colonize the gut and rectum; more than half of all cases of E. coli bacteremia can be traced back to a UTI; and physiological changes that result in bladder dysfunction and urinary catheter use raise the possibility of bacterial colonization and infection, increasing the risk of UTIs as people age [25]. Therefore, among those aged 60-90, UTI is the most common bacterial infection, with an incidence rate of 30-100 per 1000 person-years, and Escherichia coli causes the majority of UTIs [26]. Our findings are consistent with other recent research conducted in the Middle East, suggesting that E. coli is the most commonly isolated bacterium from the urinary tract; this finding is in agreement with a study from Bahrain and studies from Sri Lanka [27] and Japan, showing that K. pneumoniae is the most common resistant Enterobacteriaceae identified from respiratory. It is known for its ability to cause hospital-acquired infections and is often associated with antibiotic resistance, which the significant presence of K. pneumoniae in this figure explains. It indicates its involvement in RTIs, UTIs, and blood infections, as several factors contribute to the prevalence of E. coli and K. pneumoniae in infections. These bacteria are commonly found in the environment and can be transmitted between individuals through contaminated surfaces, food, or water. Additionally, certain risk factors, such as compromised immune systems, improper hygiene practices, or the use of invasive medical devices, can increase susceptibility to these bacterial infections [28].

The resistance rates shown in Table 3 from this study are worrying and highlight a big problem in treating infections caused by Gram-negative bacteria. Several factors contribute to the high resistance rates observed, including the overuse and misuse of antibiotics. Overprescription or inappropriate use of antibiotics can lead to the development of resistance. Inadequate dosing, incomplete courses, and use in non-bacterial infections can all contribute to this problem [29]. Gram-negative bacteria have developed various mechanisms to resist antibiotics, and these include efflux pumps that remove antibiotics from the cell, enzymes that degrade antibiotics, and changes in the bacterial cell wall that prevent antibiotics from entering the cell [30]. Bacteria can also acquire resistance genes from other bacteria through mechanisms like conjugation, transformation, and transduction, and this allows them to rapidly develop resistance to antibiotics. If the dosage of an antibiotic is too low or if it is not taken at the right intervals, bacteria may not be eradicated, allowing resistant strains to survive and multiply. Because there are more strains of E. coli that produce ESBL and AmpC and are resistant to third-generation cephalosporins, doctors are now using carbapenems more often to treat E. coli infections. Inevitably, the use of these antibiotics has led to the emergence of carbapenem-resistant strains [31]. Non-lactose fermenters like A. baumannii showed 94% susceptibility to cefotaxime, contrasting with its 51% susceptibility to piperacillin/tazobactam; this pattern mirrors findings from Karbala ICUs, where A. baumannii developed targeted resistance due to heavy piperacillin/tazobactam use (Al-Harmoo. Similarly, P. aeruginosa's 96% cefotaxime susceptibility but only 71% fosfomycin response indicates that it is necessary to administer culture-guided therapy [32]. These findings carry three key implications: First, Iraq urgently needs antimicrobial stewardship programs to curb inappropriate β-lactam and carbapenem use. Second, hospitals should implement rapid diagnostics to guide antibiotic selection, particularly for non-lactose fermenters showing divergent susceptibility patterns. Third, the regional variation in resistance profiles calls for collaborative surveillance with neighboring countries to track cross-border resistance gene spread, while without immediate intervention, Iraq risks facing untreatable infections that could destabilize its fragile healthcare system further.

Our study revealed critical resistance rates among Gram-negative isolates, with 75.5% (515/682) resistant to cefotaxime-significantly higher than rates reported in neighboring Iran (50-60%) [33] and Turkey (55-65%) [30]. Carbapenem resistance was particularly alarming (meropenem 63.8%, imipenem 58.4%), surpassing regional averages [34]. E. coli demonstrated extreme ampicillin resistance (87.4%, 284/325 isolates), and A. baumannii showed near-universal cefotaxime resistance (98.3%, 57/58), while P. aeruginosa retained relative susceptibility to amikacin (73.8%, 48/65)-consistent with Middle Eastern trends but higher than global benchmarks (CLSI 2023). These findings underscore a regional antimicrobial resistance crisis, with Iraq’s Middle Euphrates region exhibiting more severe resistance than comparable settings. Bacteria can also acquire resistance genes from other bacteria through mechanisms like conjugation, transformation, and transduction, and the result allows them to rapidly develop resistance to antibiotics. If the dosage of an antibiotic is too low or if it is not taken at the right intervals, bacteria may not be eradicated, allowing resistant strains to survive and multiply. Because there are more strains of E. coli that produce ESBL and AmpC and are resistant to third-generation cephalosporins, doctors are now using carbapenems more often to treat E. coli infections. Inevitably, such usage had resulted in the emergence of carbapenem-resistant strains [30]. The high resistance to third-generation cephalosporins (cefotaxime 75.5%, ceftazidime 71%) likely reflects widespread ESBL/AmpC production, which has driven increased carbapenem use as empirical therapy. This practice is now yielding diminishing returns, as evidenced by alarming carbapenem resistance rates in our isolates (imipenem 58.4%, meropenem 63.8%), while E. coli, historically treated with carbapenems for ESBL infections, showed 28.0% resistance to imipenem and 29.2% to meropenem, confirming the emergence of carbapenem-resistant strains under therapeutic pressure [32].

Limitations of the study

While this investigation offers useful information about the prevalence and antibiotic resistance patterns of Gram-negative bacteria in Iraq's Middle Euphrates region, certain limitations should be acknowledged. The study was conducted over a relatively short period (seven months), which may not account for seasonal variations in bacterial prevalence and resistance patterns. Additionally, the sample was restricted to hospital laboratories in four governorates, potentially limiting the generalizability of the findings to other regions or community-acquired infections. The reliance on the Vitek Compact 2 system, while efficient, may miss rare or atypical resistance mechanisms that require more advanced genomic analysis. Furthermore, the study did not investigate the underlying genetic determinants of resistance (e.g., ESBL, carbapenemase genes), which could offer additional information about resistance mechanisms. Finally, the lack of patient clinical data, such as prior antibiotic use or comorbidities, restricts the ability to correlate resistance patterns with specific risk factors. Future studies should include longer observation periods, broader geographic coverage, and molecular characterization to clarify our knowledge about antimicrobial resistance in the region. Study limitations include the lack of clinical outcome data (mortality) and molecular resistance profiling, which may affect the translation of these in vitro results to patient care.

## Conclusions

This study underscores the grave threat of multidrug-resistant Gram-negative bacterial infections across both community and healthcare settings in Iraq's Middle Euphrates region. The findings reveal alarmingly high resistance to commonly prescribed antibiotics, including widespread resistance to third-generation cephalosporins and a disturbing level of nonsusceptibility to carbapenems, which are often considered last-line agents. The predominance of pathogens such as E. coli and K. pneumoniae, coupled with the challenging resistance profiles of non-lactose fermenters like P. aeruginosa and A. baumannii, signals a critical public health crisis. To combat this escalating threat, immediate and coordinated actions are essential, including the implementation of robust antimicrobial stewardship programs to guide appropriate antibiotic use, the enhancement of laboratory capacity for rapid diagnostics, and the development of region-specific treatment guidelines based on local surveillance data. Without such interventions, the region faces the looming risk of untreatable infections, which would severely compromise patient outcomes and healthcare system stability.
